# Family Functioning and Cohesion Scale: Validation of a Short Instrument for the Assessment of Intrafamily Relations

**DOI:** 10.3390/bs14100969

**Published:** 2024-10-19

**Authors:** Vagioula Tsoutsi, Dimitris Dikeos

**Affiliations:** First Department of Psychiatry, Medical School, Eginition Hospital, National and Kapodistrian University of Athens, 11528 Athens, Greece

**Keywords:** family cohesion, intrafamily relations, family functioning, scale

## Abstract

Most available scales for assessing family relationships are lengthy. Our aim was to develop and validate the Family Functioning and Cohesion Scale (FFCS), a self-reported, short instrument consisting of 14 items. The FFCS was validated through its administration to 481 subjects via an online platform. Cronbach’s alpha was 0.85 (ranging from 0.83 to 0.86 if any one item was deleted), signifying high internal consistency. The scale can be considered as a sole factor based on its high consistency, while factor analysis produced three factors corresponding to “communication”, “anger/resentment/aggression”, and “values and beliefs”. The test–retest reliability correlation coefficient was found to be 0.88 at a 2-week interval. Regarding external validity, the correlation coefficient of the FFCS with the general functioning subscale of the McMaster Family Assessment Device (FAD) was 0.83. The high measures of consistency, reliability, and validity of the FFCS, combined with its short length, make it a most valuable tool for use by researchers as well as by professionals dealing with families in psychiatry, psychology, social work, or other related fields.

## 1. Introduction

Family is considered to be the primary source of attachment, nurturing, and socialization for humans in our society. Even though its form has changed over the centuries, it remains an important, if not the most important, social unit. There are several definitions of family. In 1940, the U.S. Census Bureau adopted a definition that has remained constant ever since. According to this traditional definition, “a family is a group of two or more people related by birth, marriage, or adoption and residing together; all such people are considered as members of one family” [1,2,3].

A family can be described as a system that, through the bonds, relationships and interactions of its members, promotes their healthy development and evolution. Family Systems Theory views the family as a unified, interconnected system, with each member serving a specific role or function within a complex, evolving network of relationships [4]. As the primary source of development and socialization, the family is one of the most important frameworks through which communication, social, and other skills are formed, skills that are necessary for human survival and development [5]. Each family member is considered to be unique, with their own personality, attitude, integrity, feelings, thoughts, and beliefs. Healthy bonds between members and the maintenance of balance and boundaries within the family are associated with the physical and mental health of its members.

Family cohesion encompasses the emotional bonding between family members, fostering a sense of belonging, support, and unity. Cohesive families are more likely to provide an environment where members feel valued, understood, and supported, which can significantly enhance their mental and emotional health. Research has shown that high levels of family cohesion are associated with positive outcomes, such as lower levels of anxiety, depression, and stress among family members [6]. A cohesive family environment promotes resilience, helping members cope with life’s challenges, whether they are related to personal development, health issues, or external stressors. For children and adolescents, a cohesive family can offer a secure base, fostering a sense of security and stability that is vital for healthy emotional and social development. In the recent research by Butler et al. [7] on the role of family, school, and peer supportive relationships in protecting the mental well-being of children and adolescents, it was found that family adult support, school adult support, and school peer support were all independently associated with mental well-being in children and adolescents, suggesting that all three sources of support are protective factors for well-being. Additionally, according to the systematic literature review of Izzo et al. [8], there is a positive relationship between happiness and family functioning across different cultures and age groups: Family dimensions, such as cohesion and communication, were found to strongly predict children’s and adolescents’ happiness.

Furthermore, family cohesion plays a protective role against risky behaviors. Adolescents from cohesive families are less likely to engage in substance abuse, delinquency, and other problematic behaviors [9]. This protective effect extends into adulthood, where individuals from cohesive family backgrounds often exhibit better social adjustment, healthier relationships, and a greater capacity for emotional regulation.

The importance of family cohesion also extends to managing chronic health conditions. In families where cohesion is strong, members are more likely to adhere to medical regimens, offer emotional support, and participate actively in the treatment of the ill member [10,11]. This supportive environment can lead to better health outcomes for individuals with chronic illnesses, such as reduced symptom severity and improved quality of life. Additionally, cohesive families are more adept at problem solving and navigating the complexities of healthcare systems, which can enhance their overall ability to manage illness and promote recovery.

Cohesion is an important concept in group dynamics theory; it arises when bonds link members of a social group to one another and to the group as a whole. “Group dynamics” refers to the internal changes within a group that trigger actions and reactions, ultimately affecting the group’s structure and its members’ behaviors [12]. Without at least some degree of cohesion, groups are expected to disintegrate as members gradually withdraw from the group. A cohesive group will be more likely to prosper over time, since it retains its members and allows them to reach goals that would elude a more incoherent aggregate [13].

The cohesion within the family is strongly related to the latter’s functioning and depends on various social determinants such as economic status, cultural identity, religious values, level of education, immigration, and social isolation. It is also influenced by large-scale events such as war, economic crises, etc., since the family is the smallest social structure and is in constant interaction with the wider community [14,15,16].

Given the significant impact of family cohesion, it is important for professionals working with families, such as psychiatrists, psychologists, social workers etc., to have a short and easy-to-use tool to assess and evaluate family cohesion in order to provide comprehensive assistance. The aim of the current study was to develop and provide such a scale that would be helpful in the daily practice of those professionals in various settings.

Most of the existing scales that examine family structure, interactions, and functioning are long. The McMaster Family Assessment Device (FAD) is a 60-item self-reported scale [17,18], the Family Environment Scale (FES) is a 90-item scale [19], and the Family Adaptability and Cohesion Evaluation Scale (FACES IV) is an 84-item scale [20]. Furthermore, most of the existing scales are based on theoretical models from the scientific fields they come from; most are based on systems theory (FACES IV) or cognitive theory (Family Communication Patterns Questionnaire) [21]. Of course, theoretical approaches are not inherently problematic. In fact, scales developed from these approaches are often scientifically robust. However, these scales tend to be lengthy and rely on underlying constructs, which may not always be suitable for quick or practical use in some professional settings.

The atheoretical approach in psychiatry was first introduced by the DSM-III and ICD-10 for the diagnosis of mental disorders. The third edition of DSM [22], published in 1980, was a true nosological revolution because it remained atheoretical. The DSM-III offered a classification which intentionally ignored the etiological models of mental disorders (since, in psychiatry, such models, while providing useful information on possible mechanisms, are still largely suggestive) and was focused on the task of providing unambiguous descriptions of these disorders by means of precise and exhaustive phenomenological diagnostic criteria [23]. In line with this atheoretical approach, the Family Functioning and Cohesion Scale (FFCS) was developed by the authors, after examination of existing scales, as a short tool to assist any professional in assessing the functioning and cohesion of families. This was done by selecting items which were most common among scales and/or were deemed relevant to the description of family characteristics.

The purpose of this paper is to present the FFCS and to document its validation.

## 2. Methods

### 2.1. Description of the Scale

The FFCS is a self-administered instrument consisting of fourteen items (see Appendix A). It focuses on the assessment of interpersonal relationships among family members, of their personal beliefs, and of their interactions and behaviors in the family.

The respondents are asked to rate their degree of agreement with each one of 14 statements on family relationships and interactions in daily life. Each item of the FFCS can be rated on a four-point scale: “Completely true”, “More true than false”, “More false than true”, and “Completely false”. For the items that contribute positively to a higher family cohesion (e.g., “We always support each other”) the highest points (i.e., a score of “3”) are given to the “completely true” statement (and a score of “0” to the “completely false”), while for the “negative” items (e.g., “There are episodes of physical violence in the family”) the score is reversed (see Scoring Rules in the Appendix A).

### 2.2. Sample and Assessment Procedures

Following the approval of the project by the institutional review ethics committee of Eginition Hospital, all subjects were approached through the network of the researchers and their collaborators, as well as through social media during the COVID-19 pandemic. On the Google Forms platform, which was used, all participants were first informed about the research nature of the project and those who gave their consent (N = 481) proceeded to complete the FFCS. To evaluate test–retest reliability, a separate group (N = 143) agreed to participate by providing their e-mail addresses, so they were assessed with the FFCS again at another time point two weeks later. The subscale for general functioning of the McMaster Family Assessment Device (FAD) was also administered to this subgroup and was used as an external validator.

### 2.3. Data Analysis

Normality was tested by examining skewness and kurtosis of the FFCS total score. Internal consistency was measured based on Cronbach’s alpha for the whole scale, as well as for all-but-one items (“alpha if item deleted”). The correlation of each item score with the total scale score (“item–total correlation”) was measured through Pearson’s correlation coefficients. Additionally, the scale was subjected to factor analysis; the extraction of the factors was based on principal component analysis with varimax rotation and the eigenvalue threshold was set at 1. Pearson’s correlation coefficients were computed for the evaluation of test–retest reliability and for the external validation of the FFCS against the McMaster Family Assessment Device (FAD); the respective correlations pertained to total scale scores as well as (for test–retest) to single-item ratings. Finally, one-way ANOVA was used to detect possible differences in the total score of the FFCS and that of each item (Bonferroni corrected) among the four country groups in which the participants declared they were residing (Greece, Rest of Europe & Australia, North/South America, Rest of the world). All assessments were performed using the Statistical Package for Social Sciences for Windows, Version 28.0 [24].

## 3. Results

The FFCS was completed by 481 participants, living in various countries around the world. The majority (57.8%) were from Greece, 22.9% were from the rest of Europe and Australia, 12.7% were from North or South America and 6.7% were from the rest of the world. Most of the participants (64.2%) were women, 54.1% were married and 95% were highly educated. About half of the participants were between 20 and 39 years old (Table 1). The mean (±SD) number of people cohabiting with the respondents was 2.81 ± 1.30, with a median of 3. In the test for assumptions of normality, skewness (−0.69) and kurtosis (+1.02) met the requirement to be between −1 and +1, although the value for kurtosis was marginal. This finding provides support for the data being considered as “normally distributed”, taking into account that it is hard for results based on Likert scales to reach normality and that parametric statistics are, anyway, robust to violations of these assumptions [25].

**Figure 1:**
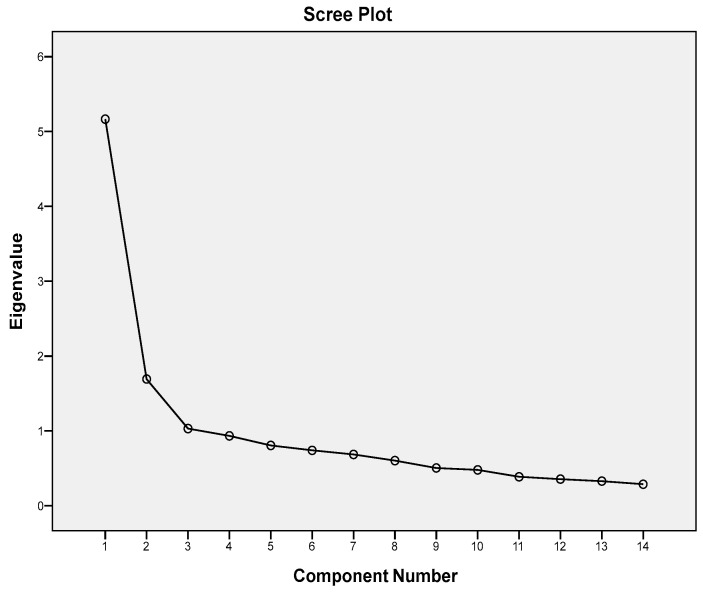
Factor Analysis Scree Plot.

Internal consistency measures of FFCS based on Cronbach’s alpha were found to be high for the total group (α = 0.85, Table 2), as well as for the group used in the test–retest reliability and external validity analysis (α = 0.88). Cronbach’s alpha remained practically unchanged when any one of the items was removed from the calculation (“alpha if item deleted” ranging from 0.83 to 0.86, Table 2). As also shown in Table 2, there were four items whose correlation coefficient with the total score was less than 0.5, but, even for these items, Cronbach’s alpha did not change significantly if any one of them was deleted. Factor analysis yielded three factors (with eigenvalues of ≥1, supported also by the scree plot shown in Figure 1) corresponding to “communication”, “anger/resentment/aggression”, and “values and beliefs”; the loadings of each item on the factors are shown in Table 3.

As for test–retest reliability, Pearson’s correlation coefficient was high (0.88 for the total score); scores for each individual item were found to be satisfactory with *p* < 0.001 for all (Table 4). More specifically, most items showed a high or moderate test–retest correlation coefficient, the highest value (r = 0.85, denoting a high correlation) was for the item “There are episodes of physical violence in the family”. Two items “We all pray together” and “There are some members of the family who impose their wishes on others” showed a low (albeit statistically significant) correlation of the test and retest ratings. The correlation coefficient between total score of the FFCS and the FAD subscore for general family functioning was high (0.83, *p* < 0.001), providing evidence of high external validity. The ANOVA of the total score of the FFCS showed that country groups did not differ among them (27.86 ± 6.04 vs. 27.75 ± 6.54 vs. 27.43 ± 8.03 vs. 27.91 ± 6.17 for the aforementioned groups, F = 0.08, df = 3,477, *p* = 0.971). For individual items, only the item “We all pray together” reached a statistically significant difference (*p* < 0.014, after Bonferroni correction). For that item the mean value (±SD) was highest for country group “rest of the world” (2.59 ± 1.21), followed by “North and South America” (1.75 ± 1.01), “Greece” (1.54 ± 0.90), and “rest of Europe and Australia” (1.37 ± 0.75); in post-hoc analysis, the difference between “rest of the world” and each one of the other groups was statistically significant, as was the difference between “North and South America” and “rest of Europe and Australia”.

## 4. Discussion

The development of the FFCS was based on the need to provide an easy-to-use tool for professionals who deal with families to assist them in evaluating family relations and providing their clients with comprehensive and integrated care according to their needs. Thus, the total score of the FFCS provides a rough estimation of the degree of family functioning and cohesion, while a low rating on any one item provides an indication that attention should be focused on that specific aspect of family life. Furthermore, the subscales (factors) that emerged in the factor analysis of the FFCS provide additional information on the three main areas of possible familial dysfunction, i.e., “communication”, “anger/resentment/aggression”, and “values and beliefs”.

The FFCS has a high internal consistency and external validity. Four of its 14 items showed a lower correlation with the scale overall, but all of them were also found to belong to the scale, since the removal of any one of them did not change the Cronbach’s alpha value substantially. The item “We all pray together” had the lowest correlation of all, probably because religious rituals do not seem to be strongly related to other aspects of family functioning. This poses the question of whether including the “We all pray together” item in the scale makes the scale more culturally sensitive: In religious cultures, praying together would be a positive factor for family cohesion; in secular cultures, not praying together does not mean anything negative for family cohesion. Despite that, we have decided to keep this item on the scale, since praying may be important to some families, and we believe that the relevant information is useful to professionals dealing with them. The other three items with relatively low item–total correlations are all associated with overt verbal and physical violence or the presence of a dominant member in the family. It seems that this pattern might also be relatively independent of other aspects of family functioning.

The external validity of the FFCS was based on its correlation with the McMaster Family Assessment Device (FAD) subscale on family general functioning. The FAD was developed in 1983 [17] and it has been used in various studies for understanding the relationship between family functioning and a wide range of mental and physical health problems across different cultures [26,27,28]. The general functioning subscale, consisting of 12 items, was specifically chosen owing to its strong conceptual alignment and similarity with the FFCS, making it an ideal benchmark for assessing the external validity of the new scale. The correlation coefficient between the FFCS and the FAD subscale was high (r = 0.83).

Based on test–retest reliability analysis, the whole FFCS and each of its items were found to be stable over time. The item with the highest correlation coefficient between the two measures was “There are episodes of physical violence in the family”. Physical violence, thus, emerges as the most temporally stable characteristic of family life, one that, if it has taken place, is not forgotten or “undone”. This is in accordance with numerous studies which show that physical violence within the family stays in the memory of all members who have experienced it and that, when it occurs, it is consistently reported by all members; it is indeed well-documented that exposure to physical violence within the family can have a lasting profound effect on the individuals involved at the physical, emotional, and psychological level, and memories of such experiences can be persistent [29,30,31].

Finally, the results of the ANOVA showed that the total score of the FFCS and all but one of its items were rated in a similar fashion regardless of geographic area of residence; the only item that differed among country groups was the item “We all pray together”. This result shows that, in general, the performance of the FFCS follows similar patterns around the world.

## 5. Limitations

This study has limitations that should be considered. First, the majority of responses were from Greece, which may limit the generalizability of the findings to other cultural and geographical contexts, since the overrepresentation of participants from a specific cultural setting might not fully capture the nuances of family cohesion in other cultural or geographic regions. Nevertheless, it must be noted that the total score of the FFCS was practically the same for all four country groups, denoting that, as regards the scale, similarities between Greece and the other three groups are stronger than any differences.

The sample composition was mainly of highly educated people, aged 20–39. High education levels could introduce bias, as family cohesion issues are often more pronounced in lower-educated families, those with lower socio-economic backgrounds, or families with migration backgrounds [32,33,34]. High education levels may, thus, affect the representativeness of the study and its findings, although it should be taken into account that the focus of the study was to establish the validity of the FFCS and its psychometric properties, rather than to determine the mean value of the population. This demographic, however, also offers a perspective on family function characteristics in young, highly educated groups.

The size of the test–retest reliability sample is relatively small, which should be considered when interpreting the results regarding the temporal stability of the scale.

Lastly, the use of a convenience sample necessitated by COVID-19 restrictions at the time of recruitment introduces potential selection bias and might decrease the robustness of the results. However, given the challenging circumstances and the need for timely data, this approach was deemed necessary to proceed with the study.

## 6. Future Research and Conclusions

The FFCS provides an easy-to-use instrument that has shown satisfactory reliability and validity. Based on the above results and limitations, future work could focus on testing this instrument in diverse populations, different cultural contexts, and different languages to further validate its generalizability, as well as testing on representative population samples. Additionally, longitudinal studies could be conducted to assess the FFCS’s sensitivity to change over time.

In conclusion, the short length and atheoretical approach of the FFCS, its excellent psychometric properties, and its three factors, each of which assesses the specific needs and problems of the families under examination, make it a useful instrument for use in research as well as for professionals dealing with families in psychiatry, psychology, social work, or any other relevant field.

## Figures and Tables

**Table 1:** Sample characteristics.

Demographics	N = 481 (%)
Female	309 (64.2%)
Married	260 (54.1%)
Employed	328 (68.2%)
High education	457 (95%)
**Age group**	
Under 20	11 (2.3%)
20–29	118 (24.5%)
30–39	110 (22.9%)
40–49	99 (20.6%)
50–59	82 (17.0%)
60–69	47 (9.8%)
70 and above	14 (2.9%)
**Country group**	
Greece	278 (57.8%)
Rest of Europe & Australia	110 (22.9%)
North/South America	61 (12.7%)
Rest of the world	32 (6.7%)

**Table 2:** Cronbach’s alpha of FFCS for whole sample (N = 481) and single-item statistics.

Cronbach’s Alpha			0.85
	Mean ± SD *	Item-total correlation	Alpha if item deleted
Total score of FFCS	27.76 ± 6.46	-	-
Items			
1. We tend to share the same beliefs (social, religious, political).	1.94 ± 0.78	0.42	0.84
2. We enjoy dining together.	2.40 ± 0.74	0.57	0.83
3. We all pray together.	0.59 ± 0.95	0.15	0.86
4. We feel better when we do not spend much time together.	2.05 ± 0.86	0.51	0.83
5. All members of the family share the same values.	1.98 ± 0.79	0.60	0.83
6. Decisions are usually taken by consensus.	2.05 ± 0.72	0.60	0.83
7. There are some members of the family who impose their wishes on others.	1.51 ± 0.91	0.39	0.84
8. We often get angry with each other.	1.63 ± 0.81	0.53	0.83
9. We often yell at each other.	1.99 ± 0.86	0.35	0.84
10. There are episodes of physical violence in the family.	2.87 ± 0.48	0.27	0.84
11. We talk openly with each other.	2.12 ± 0.81	0.63	0.83
12. We usually problem-solve together.	2.00 ± 0.77	0.67	0.82
13. We always support each other.	2.42 ± 0.69	0.67	0.83
14. We draw strength from one another.	2.21 ± 0.79	0.59	0.83

* SD = standard deviation.

**Table 3:** Factor Analysis: Rotated Component Matrix. Items loading onto each factor are shown in bold letters.

	Factors		
	1: Communication	2: Anger/Resentment/ Aggression	3: Values and Beliefs
1. We tend to share the same beliefs (social, religious, political).	0.249	0.208	**0.647**
2. We enjoy dining together.	**0.571**	0.231	0.282
3. We all pray together.	0.114	−0.195	**0.635**
4. We feel better when we do not spend much time together.	**0.419**	0.357	0.255
5. All members of the family share the same values.	0.455	0.243	**0.610**
6. Decisions are usually taken by consensus.	**0.593**	0.253	0.294
7. There are some members of the family who impose their wishes on others.	0.230	**0.579**	0.025
8. We often get angry with each other.	0.214	**0.813**	0.093
9. We often yell at each other.	0.015	**0.862**	−0.010
10. There are episodes of physical violence in the house.	0.332	**0.432**	−0.395
11. We talk openly with each other.	**0.749**	0.170	0.137
12. We usually problem-solve together.	**0.810**	0.120	0.170
13. We always support each other.	**0.821**	0.149	0.078
14. We draw strength from one another.	**0.789**	0.081	0.064

**Table 4:** Test–retest reliability.

N = 143	Test	Retest	Test-Retest
	Mean ±SD *	Mean ±SD *	Pearson Correlation (r)
Total score of FFCS	26.85 ± 6.34	27.82 ± 6.36	0.88
Items			
1. We tend to share the same beliefs (social, religious, political).	1.85 ± 0.74	1.87 ± 0.7	0.75
2. We enjoy dining together.	2.44 ± 0.67	2.31 ± 0.77	0.64
3. We all pray together.	0.31 ± 0.65	0.41 ± 0.71	0.45
4. We feel better when we do not spend much time together.	2.00 ± 0.83	2.06 ± 0.81	0.62
5. All members of the family share the same values.	1.90 ± 0.74	1.97 ± 0.73	0.73
6. Decisions are usually taken by consensus.	1.91 ± 0.73	1.91 ± 0.66	0.69
7. There are some members of the family who impose their wishes on others.	1.66 ± 0.8	1.75 ± 0.85	0.49
8. We often get angry with each other.	1.50 ± 0.83	1.70 ± 0.77	0.64
9. We often yell at each other.	1.85 ± 0.88	2.00 ± 0.82	0.70
10. There are episodes of physical violence in the house.	2.82 ± 0.51	2.81 ± 0.51	0.85
11. We talk openly with each other.	1.91 ± 0.76	2.07 ± 0.68	0.59
12. We usually problem-solve together.	1.90 ± 0.78	2.08 ± 0.65	0.60
13. We always support each other.	2.47 ± 0.65	2.54 ± 0.66	0.67
14. We draw strength from one another.	2.34 ± 0.67	2.37 ± 0.68	0.77

* SD = standard deviation, *p* < 0.001 for all.

## Data Availability

The data that support the findings of this study are available on request from the corresponding author, but restrictions apply to the availability of these data, which were used under license from the First Department of Psychiatry, Eginition Hospital, National and Kapodistrian University of Athens for the current study, and so are not publicly available. Data are, however, available from the authors upon reasonable request and with permission from the First Department of Psychiatry, Eginition Hospital, National and Kapodistrian University of Athens.

## Appendix A

### Appendix A.1. Family Functioning and Cohesion Scale (FFCS)

Please read each statement and then tick the appropriate box after considering your family’s usual practices.

1. We tend to share the same beliefs (social, religious, political) ☐ Completely true ☐ More true than false ☐ More false than true ☐ Completely false		8. We often get angry with each other ☐ Completely true ☐ More true than false ☐ More false than true ☐ Completely false
2. We enjoy dining together ☐ Completely true ☐ More true than false ☐ More false than true ☐ Completely false		9. We often yell at each other ☐ Completely true ☐ More true than false ☐ More false than true ☐ Completely false
3. We all pray together ☐ Completely true ☐ More true than false ☐ More false than true ☐ Completely false		10. There are episodes of physical violence in the family ☐ Completely true ☐ More true than false ☐ More false than true ☐ Completely false
4. We feel better when we do not spend much time together ☐ Completely true ☐ More true than false ☐ More false than true ☐ Completely false		11. We talk openly with each other ☐ Completely true ☐ More true than false ☐ More false than true ☐ Completely false
5. All members of the family share the same values ☐ Completely true ☐ More true than false ☐ More false than true ☐ Completely false		12. We usually problem-solve together ☐ Completely true ☐ More true than false ☐ More false than true ☐ Completely false
6. Decisions are usually taken by consensus ☐ Completely true ☐ More true than false ☐ More false than true ☐ Completely false		13. We always support each other ☐ Completely true ☐ More true than false ☐ More false than true ☐ Completely false
7. There are some members of the family who impose their wishes on others ☐ Completely true ☐ More true than false ☐ More false than true ☐ Completely false		14. We draw strength from one another ☐ Completely true ☐ More true than false ☐ More false than true ☐ Completely false

### Appendix A.2. Scoring Rules

For items 1–3, 5–6 and 11–14 the scoring is as follows:

Completely true: 3
More true than false: 2
More false than true: 1
Completely false: 0

For items 4 and 7–10 the scoring is as follows:

Completely true: 0
More true than false: 1
More false than true: 2
Completely false: 3
Factor 1 (communication) consists of items 2, 4, 6, 11–14
Factor 2 (anger/resentment/aggression) consists of items 7–10
Factor 3 (values and beliefs) consists of items 1, 3, 5
